# Population-based study of treatment and outcome of recurrent oesophageal or junctional cancer

**DOI:** 10.1093/bjs/znac290

**Published:** 2022-08-23

**Authors:** Marieke Pape, Pauline A J Vissers, David Bertwistle, Laura McDonald, Laurens V Beerepoot, Mark I van Berge Henegouwen, Sjoerd M Lagarde, Stella Mook, Nadia Haj Mohammad, Paul M Jeene, Hanneke W M van Laarhoven, Rob H A Verhoeven

**Affiliations:** Department of Research and Development, Netherlands Comprehensive Cancer Organization (IKNL), Utrecht, the Netherlands; Department of Medical Oncology, Cancer Centre Amsterdam, Amsterdam UMC, University of Amsterdam, Amsterdam, the Netherlands; Department of Research and Development, Netherlands Comprehensive Cancer Organization (IKNL), Utrecht, the Netherlands; Department of Surgery, Radboud University Medical Centre, Nijmegen, the Netherlands; Worldwide Health Economics and Outcomes Research, Bristol-Myers Squibb, Uxbridge, UK; Centre for Observational Research and Data Sciences, Bristol-Myers Squibb, Uxbridge, UK; Department of Medical Oncology, Elisabeth-TweeSteden Hospital, Tilburg, the Netherlands; Department of Surgery, Cancer Centre Amsterdam, Amsterdam UMC, University of Amsterdam, Amsterdam, the Netherlands; Department of Surgery, Erasmus University Medical Centre, Rotterdam, the Netherlands; Department of Radiation Oncology, University Medical Centre Utrecht, Utrecht University, Utrecht, the Netherlands; Department of Medical Oncology, University Medical Centre Utrecht, Utrecht University, Utrecht, The Netherlands; Department of Radiation Oncology, Amsterdam University Medical Centres, Amsterdam, the Netherlands; Radiotherapiegroep, Deventer, the Netherlands; Department of Medical Oncology, Cancer Centre Amsterdam, Amsterdam UMC, University of Amsterdam, Amsterdam, the Netherlands; Department of Research and Development, Netherlands Comprehensive Cancer Organization (IKNL), Utrecht, the Netherlands; Department of Medical Oncology, Cancer Centre Amsterdam, Amsterdam UMC, University of Amsterdam, Amsterdam, the Netherlands

## Abstract

**Background:**

Patients with cancer of the oesophagus or gastro-oesophageal junction have a high risk of recurrence after treatment with curative intent. The aim of this study was to analyse the site of recurrence, treatment, and survival in patients with recurrent disease.

**Methods:**

Patients with non-metastatic oesophageal or junctional carcinoma treated with curative intent between January 2015 and December 2016 were selected from the Netherlands Cancer Registry. Data on recurrence were collected in the second half of 2019. Overall survival (OS) was assessed by Kaplan–Meier methods.

**Results:**

In total, 862 of 1909 patients (45.2 per cent) for whom information on follow-up was available had disease recurrence, and 858 patients were included. Some 161 of 858 patients (18.8 per cent) had locoregional recurrence only, 415 (48.4 per cent) had distant recurrence only, and 282 (32.9 per cent) had combined locoregional and distant recurrence. In all, 518 of 858 patients (60.4 per cent) received best supportive care only and 315 (39.6 per cent) underwent tumour-directed therapy. Patients with locoregional recurrence alone more often received chemoradiotherapy than those with distant or combined locoregional and distant recurrence (19.3 per cent *versus* 0.7 and 2.8 per cent), and less often received systemic therapy (11.2 per cent *versus* 30.1 and 35.8 per cent). Median OS was 7.6, 4.2, and 3.3 months for patients with locoregional, distant, and combined locoregional and distant recurrence respectively (*P* < 0.001).

**Conclusion:**

Disease recurred after curative treatment in 45.2 per cent of patients. Locoregional recurrence developed in only 18.8 per cent. The vast majority of patients presented with distant or combined locoregional and distant recurrence, and received best supportive care.

## Teaser

In this nationwide population-based study, the vast majority of patients with recurrent disease after primary oesophageal or gastro-oesophageal junction cancer presented with distant or combined locoregional and distant recurrence. More than half of all patients received best supportive care. The prognosis of patients with recurrent disease was poor.

## Introduction

Neoadjuvant chemoradiotherapy followed by surgery is standard of care for patients with locally advanced oesophageal or gastro-oesophageal cancer^1,2^. Definitive chemoradiation is another curative treatment, most often indicated for patients with advanced disease (T4b) or for those with poor functional status or who are unwilling to undergo surgery^1,3^. In the Netherlands, follow-up focuses on quality of life and symptom control^4^. Diagnostic tests including CT and endoscopy are recommended only when patients experience disease symptoms. After treatment with curative intent, disease recurs in more than 50 per cent of patients within 3 years^5,6^. Distant recurrences are seen more often after surgery, and locoregional recurrence is more common after definitive chemoradiation^6–8^.

Median survival of patients with recurrent oesophageal cancer is 3–6 months^9–12^. This is similar to that of patients with synchronous metastatic disease, who have a median survival of 4.2 months^13^. In patients with distant recurrence at multiples sites, palliative systemic treatment or best supportive care is indicated. In those who present with limited and localized recurrence, surgical resection, definitive chemoradiation or stereotactic body radiation therapy (SBRT) may achieve long-term survival in some patients^12,14–19^.

Single-centre cohort studies^9–12,20^ have shown that patients with recurrent disease represent a diverse population in terms of treatment and survival. Such studies are prone to selection bias and have limited generalizability. A large population-based cohort study could help to better characterize treatment and survival of patients with recurrent disease in routine clinical practice. The aim of this nationwide population-based study was to assess the site of recurrence, treatment, and survival in patients with recurrent oesophageal or junctional cancer.

## Methods

### Study population

The Netherlands Cancer Registry (NCR) is a nationwide population-based cancer registry. It is based on notification of all newly diagnosed malignancies by the national automated pathology archive. Specially trained data managers routinely extract information on diagnosis, tumour stage, and treatment from medical records. Patients with adenocarcinoma or squamous cell carcinoma of the oesophagus (C15.0–C15.9) or gastro-oesophageal junction/cardia (C16.0) with recurrent disease after the diagnosis of non-metastatic disease (cT1–4A,X cNall cM0) between January 2015 and December 2016 were selected from the NCR (*Fig. S1*)^21^.

Recurrent disease was defined as outgrowth of residual disease or recurrence of previous disease after treatment with curative intent. Treatment with curative intent included resection of the tumour (oesophagectomy or (sub)total gastrectomy with or without (neo)adjuvant treatment) or definitive chemoradiation (chemotherapy with concurrent radiotherapy consisting of at least 28 fractions and/or a total radiation dose of 50 Gy or more). Metastases diagnosed during treatment with curative intent (neoadjuvant treatment or definitive chemoradiation) were considered as interval metastases, as they were diagnosed after clinical staging^22^. As not all patients underwent neoadjuvant treatment, metastases detected at or within 5 days after surgery were also considered as interval metastases.

Data on recurrence were collected in the second half of 2019. Information on vital status was available through linkage of the NCR with the Dutch Personal Records Database and updated until 1 February 2021. According to the Central Committee on Research involving Human Subjects, this type of study does not require approval from an ethics committee in the Netherlands. This study was approved by the Privacy Review Board of the NCR and the scientific committee of the Dutch Upper Gastrointestinal Cancer Group.

### Recurrence patterns

Patients were categorized according to type of recurrence (locoregional, distant, combined). Locoregional recurrence included recurrence at the site of the primary tumour or regional lymph nodes. If locoregional and distant recurrence were diagnosed within 6 weeks of each other (as treatment generally starts within 6 weeks), they were grouped as combined locoregional and distant recurrences. Time to disease recurrence was calculated from date of resection or last day of definitive chemoradiation. A subgroup analysis was undertaken in patients with recurrent oligometastatic disease. Oligometastatic disease was defined as disease limited to one organ with up to three metastases or one non-regional lymph node station^23^. Information on number of metastases per organ was available for patients with metastases limited to one organ or to the non-regional lymph nodes.

### Treatment definitions

Postrecurrence treatment was classified as resection (surgical resection of locoregional or distant recurrence), chemoradiotherapy (chemotherapy with concurrent radiotherapy independent of dose and maximum dose per fraction 2.2 Gy), SBRT for metastases (at least 10 Gy per fraction if 1 or more fractions, at least 7 Gy per fraction if 5 or more fractions, or at least 5 Gy per fraction if 12 or more fractions), systemic therapy, or best supportive care. Patients could be classified into more than one treatment group, with the exception of best supportive care which was mutually exclusive from all other treatment groups. A systemic treatment regimen comprised all chemotherapy and targeted agents that started within 3 days apart and were given until suspension^24^.

### Statistical analysis

Patient and tumour characteristics were summarized with numbers and percentages, or median (range) values, and were compared using χ^2^ test, Kurskal-Wallis test or Fisher’s exact test, as appropriate. Kaplan–Meier methods and log rank tests were used to evaluate overall survival (OS). OS was assessed from diagnosis of recurrent disease until date of death or end of follow-up. Deaths were included until 1 February 2021. *P* < 0.050 was considered statistically significant. All analyses were conducted using SAS^®^ version 9.4 (SAS Institute, Cary, NC, USA).

## Results

### Patients

In total, 2063 of 2587 patients (79.7 per cent) had treatment with curative intent, and information on follow-up was available for 1909 of 2063 patients (92.5 per cent). Patient and treatment characteristics of this population are shown in *Table S1*. Some 862 of 1909 patients were diagnosed with recurrent disease, of whom 858 were included in the study (*Fig. S1*). Median time to disease recurrence was 10.5 months (*Table 1*). Median OS after disease recurrence was 4.4 (i.q.r. 1.6–10.5) months. Recurrence according to treatment of the primary tumour is shown in *Table 2*.

**Table 1.** Characteristics of patients with disease recurrence

	All patients (*n* = 858)	Recurrence			P*
		Locoregional (*n* = 161)	Distant (*n* = 415)	Locoregional and distant (*n* = 282)	
**Sex**					0.070
M	677 (78.9%)	117 (72.7)	329 (79.3)	231 (81.9)	
F	181 (21.1)	44 (27.3)	86 (20.7)	51 (18.1)	
**Age (years), median (i.q.r.)**	68.0 (62.0–73.0)	72.0 (66.0–79.0)	67.0 (61.0–72.0)	67.0 (61.0–72.0)	<0.001†
**No. of co-morbidities**					0.029
0	388 (45.2)	56 (34.8)	204 (49.2)	128 (45.4)	
1	302 (35.2)	61 (37.9)	144 (34.7)	97 (34.4)	
≥ 2	148 (17.2)	38 (23.6)	58 (14.0)	52 (18.4)	
Unknown	20 (2.3)	6 (3.7)	9 (2.2)	5 (1.8)	
**Performance status**					0.596
0–1	325 (37.9)	70 (43.5)	152 (36.6)	103 (36.5)	
≥ 2	161 (18.8)	26 (16.1)	80 (19.3)	55 (19.5)	
Unknown	372 (43.4)	65 (40.4)	183 (44.1)	124 (44.0)	
**Time to disease recurrence (months), median (i.q.r.)**	10.5 (5.7–17.5)	13.6 (8.0–20.2)	9.4 (5.2–17.2)	10.2 (5.5–16.6)	<0.001†
**Reason for diagnosis**					0.030‡
Symptoms	677 (78.9)	119 (73.9)	320 (77.1)	238 (84.4)	
Follow-up visit	159 (18.5)	39 (24.2)	82 (19.8)	38 (13.5)	
Coincidental	10 (1.2)	1 (0.6)	8 (1.9)	1 (0.4)	
Ohter or unknown	12 (1.4)	2 (1.2)	5 (1.2)	5 (1.8)	
**Histology**					<0.001
Adenocarcinoma	659 (76.8)	101 (62.7)	343 (82.7)	215 (76.2)	
Squamous cell carcinoma	199 (23.2)	60 (37.3)	72 (17.3)	67 (23.8)	
**Treatment at primary diagnosis**					<0.001
Definitive chemoradiation	213 (24.8)	100 (62.1)	58 (14.0)	55 (19.5)	
Surgical resection with or without (neo)adjuvant treatment	645 (75.2)	61 (37.9)	357 (86.0)	227 (80.5)	
Neoadjuvant therapy at primary diagnosis					0.747‡
No neoadjuvant therapy	36 (6)	5 (8)	21 (6)	10 (4)	
Chemotherapy	55 (9)	6 (10)	30 (8)	19 (8)	
Chemoradiotherapy	554 (86)	50 (82)	306 (86)	198 (87)	
Adjuvant therapy at primary diagnosis					0.713‡
No adjuvant therapy	612 (95)	58 (95)	336 (94)	218 (96)	
Chemotherapy	30 (5)	3 (5)	18 (5)	9 (4)	
Chemoradiotherapy	3 (0)	0 (0)	3 (1)	0 (0)	
Radicality of resection					0.448‡
R0	573 (89)	52 (85)	317 (89)	204 (90)	
R1	64 (10)	7 (11)	37 (10)	20 (9)	
Unknown	8 (1)	2 (3)	3 (1)	3 (1)	
**Tumour location at primary diagnosis**					0.006‡
Cervical oesophagus	3 (0.3)	1 (0.6)	1 (0.2)	1 (0.4)	
Proximal third oesophageal	33 (3.8)	12 (7.5)	11 (2.7)	10 (3.5)	
Middle third oesophageal	100 (11.7)	30 (18.6)	36 (8.7)	34 (12.1)	
Distal third oesophageal	590 (68.8)	97 (60.2)	294 (70.8)	199 (70.6)	
Overlapping/unknown oesophageal	31 (3.6)	7 (4.3)	15 (3.6)	9 (3.2)	
Gastro-oesophageal junction/cardia	101 (11.8)	14 (8.7)	58 (14.0)	29 (10.3)	
**cT category at primary diagnosis**					0.078‡
cT1	11 (1.3)	4 (2.5)	6 (1.4)	1 (0.4)	
cT2	236 (27.5)	46 (28.6)	102 (24.6)	88 (31.2)	
cT3	554 (64.6)	99 (61.5)	278 (67.0)	177 (62.8)	
cT4	14 (1.6)	0 (0)	9 (2.2)	5 (1.8)	
cTX	43 (5.0)	12 (7.5)	20 (4.8)	11 (3.9)	
**cN status at primary diagnosis**					0.069‡
cN0	303 (35.3)	69 (42.9)	135 (32.5)	99 (35.1)	
cN1	326 (38.0)	65 (40.4)	163 (39.3)	98 (34.8)	
cN2	186 (21.7)	21 (13.0)	95 (22.9)	70 (24.8)	
cN3	32 (3.7)	5 (3.1)	15 (3.6)	12 (4.3)	
cNX	11 (1.3)	1 (0.6)	7 (1.7)	3 (1.1)	
**Tumour differentiation at primary diagnosis**					0.075
Well/moderately differentiated	343 (40.0)	72 (44.7)	166 (40.0)	105 (37.2)	
Poorly differentiated/undifferentiated	352 (41.0)	51 (31.7)	179 (43.1)	122 (43.3)	
Unknown	163 (19.0)	38 (23.6)	70 (16.9)	55 (19.5)	
**No. of sites with distant recurrence**					<0.001
0	161 (18.8)	161 (100)	–	–	
1	330 (38.5)	–	228 (54.9)	102 (36.2)	
≥ 2	367 (42.8)	–	187 (45.1)	180 (63.8)	

Values are *n* (%) unless otherwise indicated. *χ^2^ test, †Kruskal–Wallis test, and ‡Fisher’s exact test.

**Table 2:** Recurrence according to treatment of primary tumour for all patients of whom information on follow-up was available (*n* = 1909)

Treatment at primary diagnosis	No. of patients	Recurrence			
		Locoregional	Distant	Locoregional and distant	None
**Neoadjuvant chemoradiotherapy followed by surgery (without adjuvant treatment)**	1251	50 (4.0)	300 (24.0)	194 (15.5)	707 (56.5)
**Surgery alone (without (neo)adjuvant treatment)**	110	5 (4.5)	21 (19.1)	10 (9.1)	74 (67.3)
**Definitive chemoradiation**	404	101 (25.0)	59 (14.6)	55 (13.6)	189 (46.8)
Other treatments*	144	7 (4.9)	37 (25.7)	23 (16.0)	77 (53.5)

Values are *n* (%). *Surgery with (neo)adjuvant chemotherapy or surgery with neoadjuvant chemoradiotherapy and adjuvant chemo(radio)therapy.

### Pattern and timing of recurrence

**Fig. 1:**
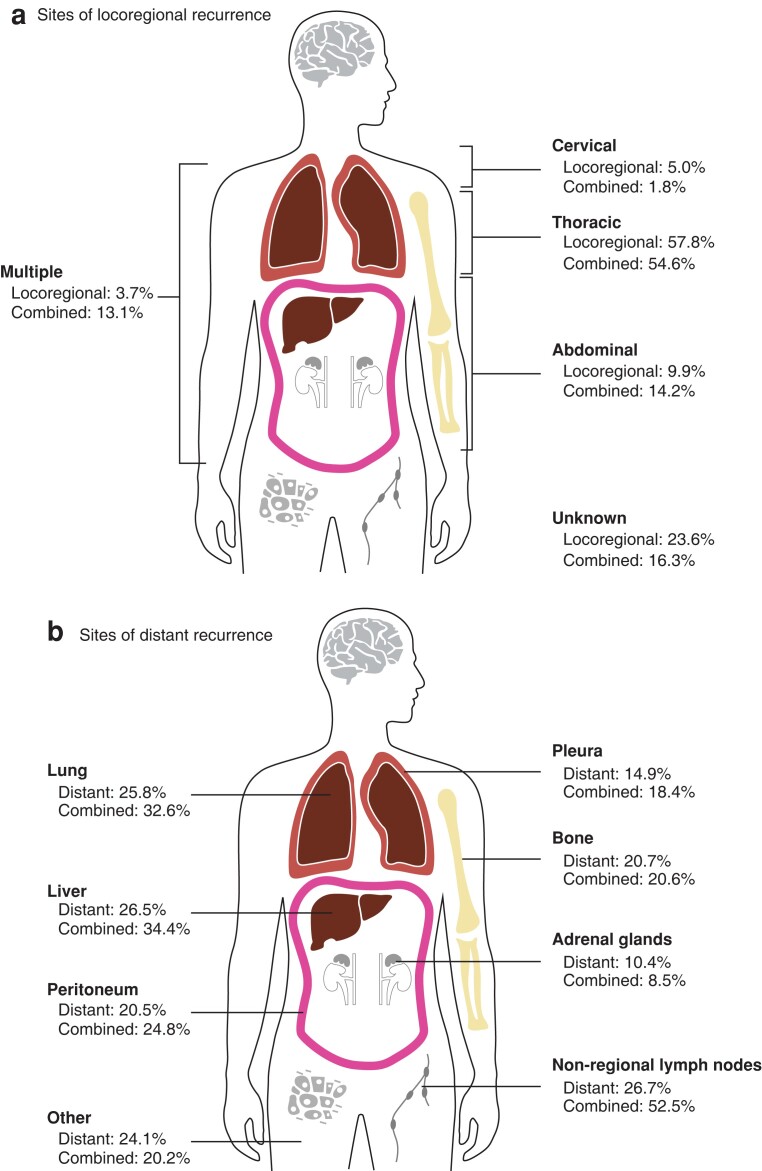
Sites of recurrence **a** Sites of locoregional recurrence in patients with locoregional recurrence or combined locoregional and distant recurrence, and **b** sites of distant recurrence in patients with distant recurrence or combined locoregional and distant recurrence.

Locoregional recurrence alone was observed in 161 of 858 patients (18.8 per cent), 415 patients (48.3 per cent) had distant recurrence only, and 282 (32.9 per cent) had combined locoregional and distant recurrence (*Table 1*). The sites of locoregional recurrence, combined locoregional and distant recurrence, and distant recurrence only are shown in *Fig. 1*. Median time to disease recurrence in patients with locoregional recurrence, distant recurrence, and combined locoregional and distant recurrence was 13.6 (i.q.r. 8.0–20.2), 9.4 (5.2–17.2), and 10.2 (5.5–16.6) months respectively (*P* < 0.001).

### Locoregional recurrence

**Fig. 2:**
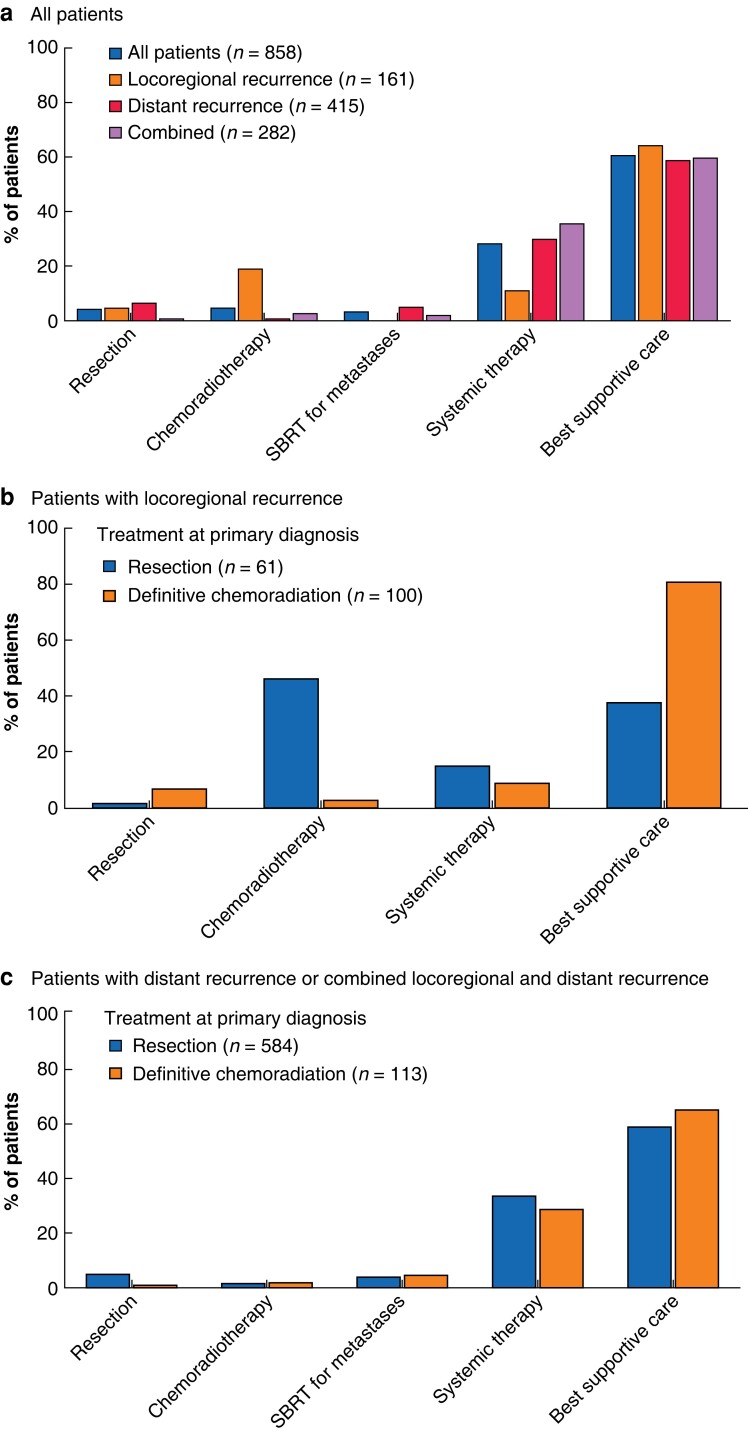
Treatment after diagnosis of recurrent disease **a** For all patients and by type of recurrence, **b** in patients with locoregional recurrence stratified by type of treatment after primary diagnosis (resection (oesophagectomy or gastrectomy) or definitive chemoradiation), and **c** in patients with distant recurrence or combined locoregional and distant recurrence stratified by type of treatment after primary diagnosis. SBRT, stereotactic body radiation therapy.

Of patients with locoregional recurrence, 5.0 per cent underwent surgery, 19.3 per cent were treated with chemoradiotherapy, 11.2 per cent had systemic therapy, and 64.6 per cent received best supportive care only after recurrence (*Fig. 2a*). Among those who received best supportive care, 27.9 per cent had radiotherapy for symptom control and 42.3 per cent received a stent for dysphagia. Nearly all patients who underwent chemoradiotherapy had a total dose of at least 50.4 Gy (90 per cent) and received carboplatin plus paclitaxel (97 per cent) (*Table S2*).

At the time of locoregional recurrence, patients who were previously treated with definitive chemoradiation were older (75.5 *versus* 67.0 years) and more often had co-morbidities (72.0 *versus* 44 per cent) than those who had surgical resection of the primary tumour. After diagnosis of locoregional recurrence, patients who had definitive chemoradiation for the primary tumour less often had chemoradiotherapy (3.0 *versus* 46 per cent; *P* < 0.001) and more often received best supportive care (81.0 *versus* 38 per cent; *P* < 0.001) than patients who underwent oesophagectomy or gastrectomy for the primary tumour (*Fig. 2b*). In the subgroup of patients with locoregional recurrence who received best supportive care, those who had definitive chemoradiation for the primary tumour less often underwent radiotherapy for symptom control (15 per cent), but more often received a stent (52 per cent) compared with patients who had surgery for the primary tumour (radiotherapy for symptom control 74 per cent, stent 9 per cent).

**Fig. 3:**
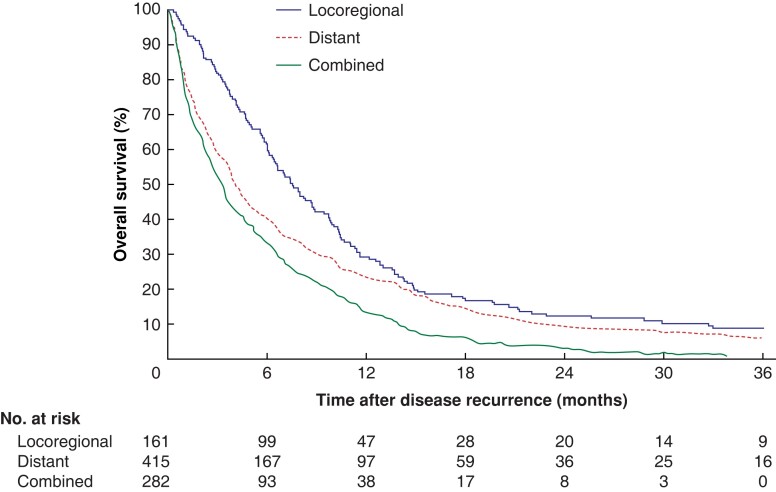
Overall survival of patients with locoregional recurrence, distant recurrence or locoregional and distant recurrence *P* < 0.001 (log rank test).

**Fig. 4:**
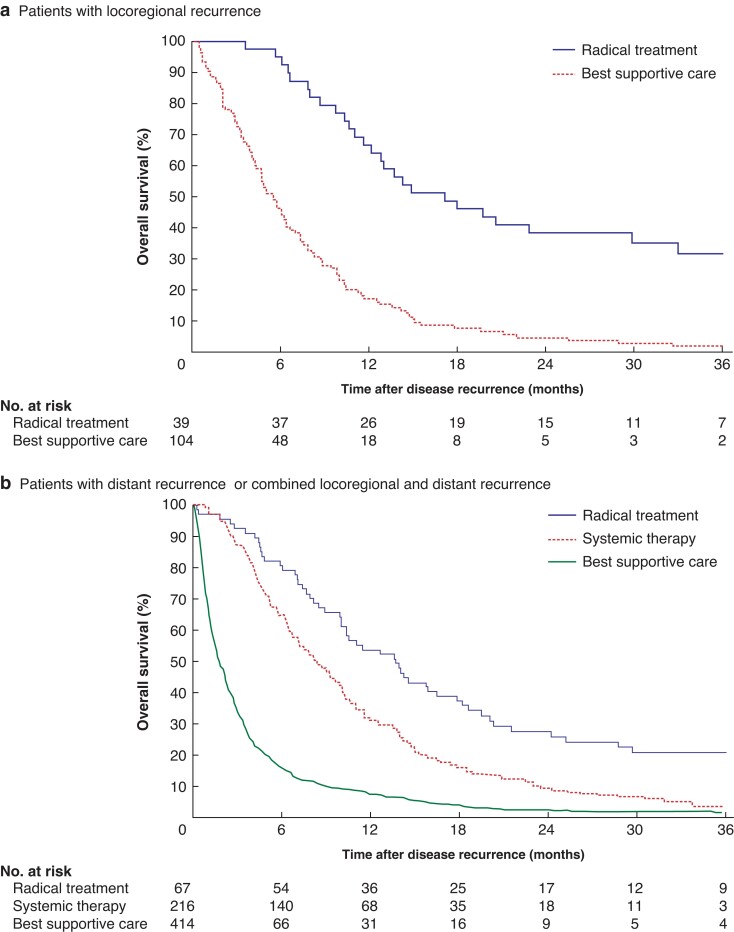
Overall survival of patients with locoregional recurrence, or combined locoregional and distant recurrence by type of treatment after recurrence **a** Locoregional recurrence and **b** combined locoregional and distant recurrence, **a**,**b***P* < 0.001 (log rank test).

Median OS for patients with locoregional recurrence was 7.6 (i.q.r. 3.9–13.7) months (*Fig. 3*). It was 17.2 (10.4–44.9) and 5.6 (2.9–9.9) months after receiving radical treatment (chemoradiotherapy, resection, and/or SBRT for metastases) and best supportive care respectively (*P* < 0.001) (*Fig. 4a*). In patients with locoregional recurrence, median survival was longer for those who had surgery for the primary tumour than for patients who had definitive chemoradiation: 11.1 (7.1–19.8) *versus* 5.9 (3.1–9.9) months (*P* < 0.001) (*Fig. S2*).

### Distant recurrence or combined locoregional and distant recurrence

Of patients with distant recurrence, 6.5 per cent underwent resection of the distant metastasis, 0.7 per cent had chemoradiotherapy, 5.3 per cent received SBRT for metastases, 30.1 per cent underwent systemic therapy, and 59.0 per cent received best supportive care only after recurrence (*Fig. 2a*). Among patients with combined locoregional and distant recurrence, 0.7 per cent underwent resection, 2.8 per cent received chemoradiotherapy, 2.1 per cent had SBRT for metastases, 35.8 per cent underwent systemic therapy, and 59.9 per cent received best supportive care only after recurrence. The majority of patients who had systemic therapy received doublet therapy (73.5 per cent), most commonly capecitabine and oxaliplatin (47.8 per cent) followed by carboplatin and paclitaxel (18.1 per cent) (*Table S2*). There were no differences in the treatment of recurrence (distant only or combined locoregional and distant) between patients who at primary diagnosis underwent resection of the primary tumour and those who had definitive chemoradiation after diagnosis and developed distant or combined locoregional and distant recurrence (*Fig. 2c*).

Median OS was 4.2 (i.q.r. 1.4–11.1) and 3.3 (1.1–7.7) months for patients with distant or combined locoregional and distant recurrence respectively (*Fig. 3*). As similar treatment patterns were observed for patients with distant recurrence or combined locoregional and distant recurrence, these patients were combined for the following survival analysis. Among patients with distant recurrence or combined locoregional and distant recurrence, median OS was 13.7 (7.1–25.2), 8.3 (4.5–14.2), and 1.8 (0.8–3.9) months for those receiving radical treatment, systemic therapy, and best supportive care respectively (*P* < 0.001) (*Fig. 4b*).

### Oligometastatic disease

Of the 697 patients with distant or combined locoregional and distant recurrence, metastases were limited to a single site in 330 (47.3 per cent) (*Table 1*). The number of metastases was known in 181 of these patients (54.9 per cent), and 143 (79.0 per cent) were considered to have oligometastatic disease. Most common sites were brain (28 patients), lung (24 patients), bone (24 patients), liver (22 patients), and non-regional lymph nodes (19 patients). Among patients with oligometastatic disease, 110 (76.9 per cent) had distant recurrence, and 33 (23.1 per cent) had combined locoregional and distant recurrence.

Some 16.8 per cent of patients with oligometastatic disease underwent metastasectomy, 4.2 per cent had chemoradiotherapy, 14.7 per cent received SBRT for metastases, 28.0 per cent underwent systemic therapy, and 39.9 per cent received best supportive care only (*Table S3*). Median OS of patients with oligometastatic disease was 10.2 (i.q.r. 4.5–21.0) months overall. It was 15.8 (8.9–29.7), 12.1 (7.1–22.9), and 4.7 (2.3–11.7) months for patients receiving radical treatment, systemic therapy, and best supportive care respectively.

## Discussion

The present study has shown that patients who develop disease recurrence after curative treatment have a poor prognosis. More than half of all patients received best supportive care and long-term survival was limited to a few patients.

A previous Dutch study^10^ of patients with recurrent oesophageal cancer after surgery between 1993 and 2010 reported a median survival of 3.0 months. Median survival was 4.4 months in the present study, indicating a marginal improvement since 2010. This may be explained by the higher proportion of patients with locoregional recurrence and tumour-directed therapy in the present study.

In the Netherlands, follow-up after treatment with curative intent is recommended 3 weeks after treatment, every 3 months in the first year, and half yearly thereafter until 5 years after treatment^4^. Radiological tests or endoscopies to detect recurrent disease are recommended only when patients experience disease symptoms. This strategy may have an effect on the proportion of patients diagnosed with locoregional *versus* distant recurrence. Diagnosis is delayed compared with routine diagnostic testing during follow-up, which could result in lead time bias in survival time after recurrence. Recently, an international multicentre study^25^ reported that radiological follow-up (defined as minimum annual CT for 3 years after surgery) was associated with improved survival for patients with localized disease and those who underwent surgery only. Additionally, radiological follow-up was associated with a higher proportion of patients receiving systemic therapy after recurrence^25^. Whether more intensive surveillance strategies, for example radiological tests and/or endoscopies, would have an impact on the type of recurrence (locoregional *versus* distant), and consequently on treatment and survival, after recurrent disease in the Netherlands is unknown.

In the present study, patients with distant or combined locoregional and distant recurrence treated with systemic therapy had similar survival (8.3 months) to patients with synchronous metastatic disease (7.5 months)^24^. First-line chemotherapy combined with anti-programmed cell death protein 1 (anti-PD-1) therapy is a new standard of care, and expected to improve survival of patients with human epidermal growth factor receptor 2-negative oesophagogastric carcinoma and high programmed death-ligand 1 (PD-L1) expression^26,27^. Additionally, anti-PD-1 therapy is indicated in the adjuvant setting in a subset of patients after neoadjuvant chemoradiotherapy and surgery, based on the results of the CheckMate 577 study^28^. However, it remains uncertain whether anti-PD-1 therapy is of benefit in patients with recurrent disease as these patients might have already received anti-PD-1 therapy in the adjuvant setting.

Survival of patients receiving best supportive care was very poor. Reasons for refraining from systemic therapy were unknown, but it could be that the physical state of patients precluded this treatment, or because of patient preference and perceived short life expectancy. Currently, systemic therapy is not advised for patients near the end of life as it is associated with poorer quality of life^29–31^. Therefore, it could be speculated that not giving systemic therapy was the right decision. The results of the present study emphasize the importance of high-quality palliative care for patients with recurrent disease.

Patients who received radical treatment, for both locoregional and distant recurrence, survived for longer than those who received a different type of treatment. Salvage oesophagectomy is an option for a well selected subgroup of patients after definitive chemoradiation (7 per cent among patients with locoregional recurrence in the present study). Although long-term survival can be achieved, salvage oesophagectomy is associated an increased risk of postoperative morbidity and mortality^32,33^.

Radical treatment was more common among patients with oligometastatic disease than those with distant recurrence. This is supported by data from recent cohort studies^17–19^ on oligometastatic disease that reported improved survival for these patients. However, a randomized study has not been performed. In the present study, 39.9 per cent of patients with oligometastatic disease received best supportive care only, and the number of patients who received both systemic and local treatment was limited (2.8 per cent).

In contrast to previous single-centre cohort studies^11,12,34^, the present analysis included patients who developed recurrence after either surgical resection or definitive chemoradiation. Definitive chemoradiation is considered a treatment with curative intent as this is an alternative to surgical resection^1,35^. Systemic therapy is not the preferred method for palliation of dysphagia in patients with locoregional recurrence, and reirradiation or resection of a locoregional recurrence is often not possible in patients who received definitive chemoradiation^36,37^. Of these patients, a higher proportion received best supportive care for recurrent disease, and survival was poorer than that among patients who underwent surgery. The shorter postrecurrence survival may be explained by poorer physical functioning of patients who received definitive chemoradiation compared with patients who underwent surgery for the primary tumour. At the time of disease recurrence, patients who had undergone definitive chemoradiation for the primary tumour were older and had more co-morbidities.

A strength of the present study is that a nationwide cohort was included with information on current practice for recurrent disease. However, some variables were reported incompletely, for example performance status. It was also unknown whether patients presented with solitary locoregional recurrence or had oligometastatic recurrence. For patients with locoregional recurrence, it was unknown whether the recurrence developed at the anastomotic site. Finally, no subgroup analyses according to type of recurrence and treatment were undertaken owing to the limited sample size.

This population-based study has shown that patients with recurrent disease after treatment for oesophageal or gastro-oesophageal junctional cancer have a poor prognosis, especially in the setting of distant or combined locoregional and distant recurrence. Patients who underwent radical treatment had the longest survival, and future research should assess whether more patients may benefit from tumour-directed therapy. In addition, there is a need for novel treatments for patients with poor performance status to improve their outcome.

## Supplementary Material

znac290_Supplementary_Data
