# Supplementary material for: Population-based study of treatment and outcome of recurrent oesophageal or junctional cancer
Source: Br J Surg. 2022 Aug 23;109(12):1264–73. doi: 10.1093/bjs/znac290 (PMC10364682; doi:10.1093/bjs/znac290)
Supplement: znac290_Supplementary_Data [file znac290_supplementary_data.zip › Supplementary_Figure_2.docx]

**Figure S2. Overall survival of patients with locoregional recurrence by type of treatment after primary diagnosis.**
