# Supplementary material for: Population-based study of treatment and outcome of recurrent oesophageal or junctional cancer
Source: Br J Surg. 2022 Aug 23;109(12):1264–73. doi: 10.1093/bjs/znac290 (PMC10364682; doi:10.1093/bjs/znac290)
Supplement: znac290_Supplementary_Data [file znac290_supplementary_data.zip › Supplementary_Table_1.docx]

**Table S1. Characteristics of patients with oesophageal or junctional cancer who received treatment with curative intent according to histology.**

|  | **All patients** (n=1909) | **Adenocarcinoma** (n=1395) | **Squamous cell carcinoma** (n=514) | **P-value** |
| --- | --- | --- | --- | --- |
| **Sex**, n (%) |  |  |  | <.001^1^ |
| Male | 1442 (75.5%) | 1176 (84.3%) | 266 (51.8%) |  |
| Female | 467 (24.5%) | 219 (15.7%) | 248 (48.2%) |  |
| **Age** |  |  |  | 0.003^2^ |
| Median (IQR) | 67.0 (60.0-72.0) | 66.0 (60.0-72.0) | 67.0 (62.0-72.0) |  |
| **Comorbidities**, n (%) |  |  |  | 0.148^1^ |
| 0 | 903 (47.3%) | 676 (48.5%) | 227 (44.2%) |  |
| 1 | 604 (31.6%) | 442 (31.7%) | 162 (31.5%) |  |
| ≥2 | 331 (17.3%) | 230 (16.5%) | 101 (19.6%) |  |
| Unknown | 71 (3.7%) | 47 (3.4%) | 24 (4.7%) |  |
| **Performance status**, n (%) |  |  |  | 0.001^1^ |
| 0-1 | 1531 (80.2%) | 1141 (81.8%) | 390 (75.9%) |  |
| ≥2 | 113 (5.9%) | 67 (4.8%) | 46 (8.9%) |  |
| Unknown | 265 (13.9%) | 187 (13.4%) | 78 (15.2%) |  |
| **Treatment at primary diagnosis**, n (%) |  |  |  | <.001^1^ |
| Definitive chemoradiation | 404 (21.2%) | 169 (12.1%) | 235 (45.7%) |  |
| Surgical resection with or without (neo)adjuvant treatment | 1505 (78.8%) | 1226 (87.9%) | 279 (54.3%) |  |
| *Neoadjuvant therapy at primary diagnosis, n (%)* |  |  |  | <.001^3^ |
| No neoadjuvant therapy | 111 (7.4%) | 91 (7.4%) | 20 (7.2%) |  |
| Chemotherapy | 119 (7.9%) | 116 (9.5%) | 3 (1.1%) |  |
| Chemoradiotherapy | 1275 (84.7%) | 1019 (83.1%) | 256 (91.8%) |  |
| *Adjuvant therapy at primary diagnosis, n (%)* |  |  |  | <.001^3^ |
| No adjuvant therapy | 1428 (94.9%) | 1149 (93.7%) | 279 (100.0%) |  |
| Chemotherapy | 74 (4.9%) | 74 (6.0%) | 0 (0.0%) |  |
| Chemoradiotherapy | 3 (0.2%) | 3 (0.2%) | 0 (0.0%) |  |
| *Radicality of resection, n (%)* |  |  |  | 0.154^3^ |
| R0 | 1392 (92.5%) | 1132 (92.3%) | 260 (93.2%) |  |
| R1 | 91 (6.0%) | 79 (6.4%) | 12 (4.3%) |  |
| R2 | 1 (0.1%) | 1 (0.1%) | 0 (0.0%) |  |
| Unknown | 21 (1.4%) | 14 (1.1%) | 7 (2.5%) |  |
| **Tumour location**, n (%) |  |  |  | <.001^3^ |
| Cervical oesophagus | 9 (0.5%) | 1 (0.1%) | 8 (1.6%) |  |
| Proximal third oesophageal | 90 (4.7%) | 2 (0.1%) | 88 (17.1%) |  |
| Middle third oesophageal | 249 (13.0%) | 49 (3.5%) | 200 (38.9%) |  |
| Distal third oesophageal | 1281 (67.1%) | 1095 (78.5%) | 186 (36.2%) |  |
| Overlapping/Unknown oesophageal | 63 (3.3%) | 31 (2.2%) | 32 (6.2%) |  |
| Gastro-oesophageal junction/Cardia | 217 (11.4%) | 217 (15.6%) | 0 (0.0%) |  |
| **cT-stage**, n (%) |  |  |  | 0.001^1^ |
| cT1 | 68 (3.6%) | 47 (3.4%) | 21 (4.1%) |  |
| cT2 | 572 (30.0%) | 417 (29.9%) | 155 (30.2%) |  |
| cT3 | 1158 (60.7%) | 868 (62.2%) | 290 (56.4%) |  |
| cT4 | 25 (1.3%) | 14 (1.0%) | 11 (2.1%) |  |
| cTX | 86 (4.5%) | 49 (3.5%) | 37 (7.2%) |  |
| **cN-stage**, n (%) |  |  |  | 0.195^1^ |
| cN0 | 814 (42.6%) | 591 (42.4%) | 223 (43.4%) |  |
| cN1 | 697 (36.5%) | 500 (35.8%) | 197 (38.3%) |  |
| cN2 | 324 (17.0%) | 251 (18.0%) | 73 (14.2%) |  |
| cN3 | 48 (2.5%) | 37 (2.7%) | 11 (2.1%) |  |
| cNX | 26 (1.4%) | 16 (1.1%) | 10 (1.9%) |  |
| **Tumour differentiation**, n (%) |  |  |  | <.001^1^ |
| Well/moderate | 852 (44.6%) | 599 (42.9%) | 253 (49.2%) |  |
| Poorly/undifferentiated | 662 (34.7%) | 536 (38.4%) | 126 (24.5%) |  |
| Unknown | 395 (20.7%) | 260 (18.6%) | 135 (26.3%) |  |
| **Type of recurrence**, n (%) |  |  |  | <.001^1^ |
| Locoregional recurrence | 163 (8.5%) | 103 (7.4%) | 60 (11.7%) |  |
| Distant recurrence | 417 (21.8%) | 345 (24.7%) | 72 (14.0%) |  |
| Combined locoregional and distant recurrence | 282 (14.8%) | 215 (15.4%) | 67 (13.0%) |  |
| No recurrence | 1047 (54.8%) | 732 (52.5%) | 315 (61.3%) |  |
| ^1^Chi-Square p-value; ^2^Kruskal-Wallis p-value; ^3^Fisher Exact p-value | | | | |
