# Supplementary material for: Population-based study of treatment and outcome of recurrent oesophageal or junctional cancer
Source: Br J Surg. 2022 Aug 23;109(12):1264–73. doi: 10.1093/bjs/znac290 (PMC10364682; doi:10.1093/bjs/znac290)
Supplement: znac290_Supplementary_Data [file znac290_supplementary_data.zip › Supplementary_Table_2.docx]

**Table S2. Details of type of treatment after disease recurrence.**

| ***Systemic therapy*** | **All patients**  (n=244) | **Recurrence** | | |
| --- | --- | --- | --- | --- |
|  |  | **Locoregional**  (n=18) | **Distant**  (n=125) | **Locoregional and distant**  (n=101) |
| **General** |  |  |  |  |
| Monotherapy | 11 (4.5%) | 0 (0.0%) | 7 (5.6%) | 4 (4.0%) |
| Doublet | 181 (74.2%) | 15 (83.3%) | 92 (73.6%) | 74 (73.3%) |
| Triplet | 21 (8.6%) | 1 (5.6%) | 12 (9.6%) | 8 (7.9%) |
| Trastuzumab-containing regimen | 26 (10.7%) | 1 (5.6%) | 12 (9.6%) | 13 (12.9%) |
| Non-trastuzumab containing targeted therapy^1^ | 5 (2.0%) | 1 (5.6%) | 2 (1.6%) | 2 (2.0%) |
| **Specification^2^** |  |  |  |  |
| Capecitabine and oxaliplatin (CAPOX) | 116 (47.5%) | 8 (44.4%) | 63 (50.4%) | 45 (44.6%) |
| Carboplatin and paclitaxel | 44 (18.0%) | 3 (16.7%) | 22 (17.6%) | 19 (18.8%) |
| Capecitabine, oxaliplatin and trastuzumab | 19 (7.8%) | 1 (5.6%) | 7 (5.6%) | 11 (10.9%) |
| 5-FU, leucovorin and oxaliplatin (FOLFOX) | 16 (6.6%) | 2 (11.1%) | 5 (4.0%) | 9 (8.9%) |
| Epirubicin, oxaliplatin and capecitabine (EOX) | 14 (5.7%) | 0 (0.0%) | 10 (8.0%) | 4 (4.0%) |
| Other | 35 (14.3%) | 4 (22.2%) | 18 (14.4%) | 13 (12.9%) |
| ***Chemoradiotherapy*** | **All patients**  (n=42) | **Recurrence** | | |
|  |  | **Locoregional**  (n=31) | **Distant**  (n=3) | **Locoregional and distant**  (n=8) |
| **Total radiation dose** |  |  |  |  |
| < 41.4 Gy | 2 (4.8%) | 2 (6.5%) | 0 (0%) | 0 (0%) |
| 41.4 Gy-50.4 Gy | 4 (9.5%) | 1 (3.2%) | 0 (0%) | 3 (37.5%) |
| ≥50.4 Gy | 36 (85.7%) | 28 (90.3%) | 3 (100%) | 5 (62.5%) |
| **Chemotherapy regimen** |  |  |  |  |
| Carboplatin and paclitaxel | 40 (95.2%) | 30 (96.8%) | 3 (100%) | 7 (87.5%) |
| Capecitabine and cisplatin | 1 (2.4%) | 0 (0%) | 0 (0%) | 1 (12.5%) |
| 5-FU and cisplatin | 1 (2.4%) | 1 (3.2%) | 0 (0%) | 0 (0%) |
|  |  | **Recurrence** | | |
| ***Resection*** | **All patients**  (n=37) | **Locoregional**  (n=8) | **Distant**  (n=27) | **Locoregional and distant**  (n=2) |
| **Type of resection** |  |  |  |  |
| Salvage resection | 7 (18.1%) | 7 (87.5%) | 0 (0.0%) | 0 (0.0%) |
| Gastric tube resection | 1 (2.7%) | 1 (12.5%) | 0 (0.0%) | 0 (0.0%) |
| Metastasectomy | 29 (78.3%) | 0 (0.0%) | 27 (100.0%) | 2 (100.0%) |

^1^All non-trastuzumab targeted containing regimens consisted of paclitaxel and ramucirumab.

^2^Specification of regimens are included if administered in at least 5.0% of patients.
